# Double burden of malnutrition among ever-married women in Bangladesh: a pooled analysis

**DOI:** 10.1186/s12905-019-0725-2

**Published:** 2019-01-31

**Authors:** Tania Sultana Tanwi, Sayan Chakrabarty, Syed Hasanuzzaman

**Affiliations:** 1 Department of Economics, Shahjalal University of Science & Technology, Sylhet Kumargaon, Sylhet, 3114 Bangladesh; 2 University of Southern Queensland, Springfield, QLD 4300 Australia; 3 Department of Economics, Shahjalal University of Science & Technology, Sylhet Kumargaon, Sylhet, 3114 Bangladesh

**Keywords:** Double burden of malnutrition, Women, Bangladesh, Pooled analysis

## Abstract

**Background:**

Evidences show that the burden of overweight and obesity is escalating in developing countries with predominant burden of underweight. The coexistence of underweight and overweight/obesity is known as double burden of malnutrition. Recent scanty studies confirmed that Bangladesh is currently experiencing augmented overweight and obesity as well as abating underweight. The present study aimed at assessing the changes of prevalence of overweight/obesity and underweight from 2004 to 2014 and investigated the socio-demographic correlates of being overweight/obese and underweight among ever-married women age 15–49 years.

**Methods:**

Data were collected from four consecutive Demographic and Health Surveys conducted in Bangladesh in 2004 (*N* = 11,173), 2007 (*N* = 10,993), 2011 (*N* = 17,749), 2014 (*N* = 17,690). Multinomial logistic regression model has been used to determine association between different socio-demographic predictors with overweight/obesity and underweight among ever-married women age 15–49 years considering normal weight as reference category.

**Results:**

The prevalence of underweight decreased by 43.2% (from 32.2% in 2004 to 18.3% in 2014) and 130.5% increase in overweight and obesity (from 10.5% in 2004 to 24.2% in 2014) were found over the ten years period. Age, educational status, wealth index and year were positively associated with overweight and obesity and negatively associated with underweight. Also, ‘not being married’ status for rural women were positively associated with underweight and negatively associated with overweight and obesity. Rural women were less likely to be overweight and obese (OR = 0.7, 95% CI: 0.7–0.8) while more likely to be underweight (OR = 1.1, 95% CI: 1.1–1.2) relative to urban women respectively. The likelihood of being overweight and obese was 4.5 times (95% CI: 4.1–4.9) higher among women who were in richest quintile compared to poorest women. They were also less likely to be underweight (OR = 0.4, 95% CI: 0.3–0.4) relative to same reference category.

**Conclusion:**

The double burden of malnutrition is evidently prevailing in Bangladesh. Over the ten years period, overweight and obesity has been raised tremendously but underweight did not fall significantly. This study suggests that strategies for preventing both underweight and overweight/obesity simultaneously among reproductive women need to be implemented considering regional context and their socioeconomic status (SES).

## Background

Developing countries have been undergoing a numerous form of transitions including epidemiological transition which is a transformation process of changing pattern of diseases [1]. Because of the elevated prevalence of overweight/obesity and predominant prevalence of underweight, many low- to middle-income countries are addressing both extreme cases of malnutrition [2, 3]. The situation of escalating prevalence of overweight and obesity with persisting prevalence of underweight is called double burden of malnutrition [4–6].Rising economic development, rapid urbanization, changes in food production, dietary pattern and physical activity are accountable for nutrition transition [5, 7–9]. Both types of malnutrition; underweight and overweight are associated with different forms of adverse health problem. One the one hand, overweight and obesity is associated with many non-communicable diseases (NCDs), such as type 2 diabetes, cardiovascular disease,, respiratory problem, and many others [10–13].On the other hand, undernutrition is associated with variety of communicable diseases (CDs) as well as low birth weight, preterm birth, mental health impairment, increased risk of infant mortality, higher risk of early mortality [14–17].The World Health Organization (WHO) and the International Obesity Task Force (IOTF) have found both underweight and overweight/obesity as one of the emerging epidemic among top ten future global health problems in developing countries [18, 19].

Evidence shows that in most developing countries, prevalence of overweight surpassed prevalence of underweight among women in both urban and rural areas [7]. Overweight and obesity with NCDs are continuously increasing in Asia though this region is top ranked having highest number of underweight people, with 163 million and 300 million in East Asia and South Asia respectively [20]. According to the Balarajan and Villamor (2009), the prevalence of underweight declined modestly in Nepal and India but substantially in Bangladesh during the period of 1996 to 2006. By this time, the prevalence of overweight and obesity inclined from 2.7 to 8.9% in Bangladesh; from 1.6 to 10.1% in Nepal and from 10.6 to 14.8% in India [21]. Several other studies confirmed that Bangladesh is currently facing double burden of malnutrition. The prevalence of underweight decreased substantially from 1996 to 2011 whereas the prevalence of overweight and obesity increased at an alarming rate over the same period [22–24]. Both types of malnutrition; underweight and overweight have been emerged as severe health problems which mean that country is in verge of double disease burden. A little knowledge is known about this new epidemic of double burden of malnutrition. However, the correlation of child’s health with maternal health made us concern about women’s health pertaining this specific issue [17, 25]. The present study aimed to explore the changes of prevalence of underweight and overweight/obesity over the ten years period using pooled data from four successive Bangladesh Demographic and Health Surveys (BDHS) conducted from 2004 to 2014. To our knowledge, no such study has been reported including the most updated survey, 2014 BDHS. We also investigated the socio-demographic determinants of being underweight and overweight/obese. The outcomes of this study will help policy-makers to grasp paradoxical situation between policies to combat existing underweight and accelerating overweight and obesity.

## Methods

Data were collected from the Bangladesh Demographic and Health Survey (BDHS). The nationally representative surveys covered information on demographic characteristics, family planning, maternal and children’s health and nutritional status. National Institute of Population Research and Training (NIPORT) of the Ministry of Health and Family Welfare (MOHFW) supervised the all BDHS surveys. The present study used consecutive surveys of BDHS 2004, 2007, 2011, 2014 for pooled analysis [26–29]. Data have been extracted from ‘Woman’s Questionnaire’. The ‘Woman’s Questionnaire’ usually collects information from ever-married women age 15–49 years. All surveys were conducted based on two-stage stratified sampling technique. In the first stage, sample clusters were selected from main sampling frame which was constructed from Population and Housing Census (PHC) of Bangladesh. In the second stage, a systematic sample of 30 households was selected for each cluster.

### Data collection

**Fig. 1:**
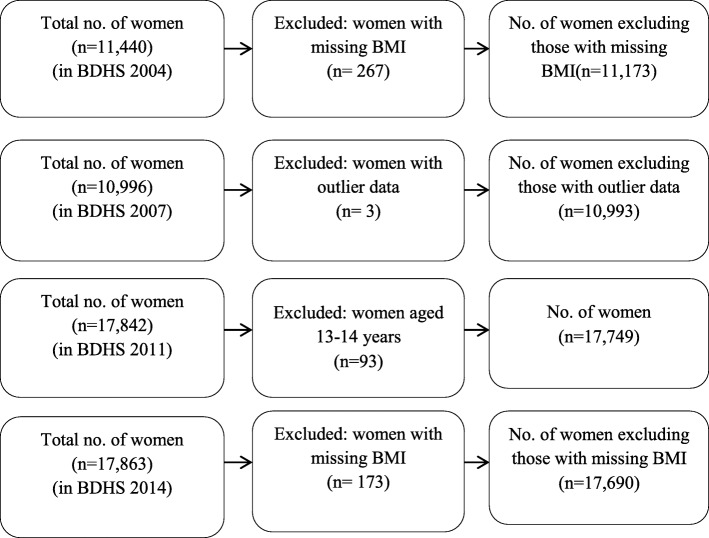
Flow chart of data extraction from BDHS 2004, BDHS 2007, BDHS 2011, and BDHS 2014

Trained personnel were appointed to measure anthropometric data of height and weight using a standardized procedure (A standardized measuring board with accuracy to 0.1 cm has been used to measure height and solar powered UNICEF electronic scale with accuracy to 0.1 kg has been used to measure weight). First, we extracted data from women file of respective year of BDHS and also extracted required variables. Missing values and outlier data of considered variables had been checked. Women age 13–14 years were excluded from BDHS 2011 for keeping similar age range of women with other surveys. The pregnant women were excluded from this study. Overall, data (*N* = 57,605) of ever-married non-pregnant women age 15–49 year were collected from 2004 (*N* = 11,173), 2007(*N* = 10,993), 2011 (*N* = 17,749), 2014 (*N* = 17,690) in this final analysis Fig. 1

### Variables

In this study, body mass index (BMI) was the dependent variable indicated as women’s weight status. BMI was calculated by dividing body weight (kg) by height squared (m^2^). According to World Health Organization (WHO), BMI was divided into four categories as: underweight (BMI < 18.5 kg/m^2^), normal weight (18.5 ≤ BMI < 25 kg/m^2^), overweight (25 ≤ BMI < 30 kg/m^2^) and obese (BMI ≥ 30 kg/m^2^) [30]. Overweight and obesity were combined as one category. The predictor variables (categories are shown in parenthesis) used in this study as: age (15–19, 20–29, 30–39, 40–49 years); Place of residence (Urban/Rural) (urban, rural); education (no education, primary, secondary, higher); parity (no children, 1–2 children, 3–4 children, 5+ children); marital status (married, widowed, divorced, separated); wealth status of a household was represented by wealth index (poorest, poorer, middle, richer, richest).The BDHSs constructed wealth index variable by principle component analysis (PCA) using data on selected household assets [29].

### Statistical analysis

Analysis of data started with descriptive statistics to know the relationship between socio-demographic characteristics and BMI among Bangladeshi women (Table 1). Multinomial logistic regression analysis was performed to evaluate the association between socio-demographic factors and BMI among women. BMI was categorized as underweight, normal weight and overweight/obesity. Underweight and overweight/obesity were compared to the reference category normal weight respectively. Results of multinomial logistic regression analysis were presented by odds ratio (OR) at 95% confidence intervals (CIs). Statistical significance was set at a *P*-value of 0.001. The overall *P*-value for the multinomial logistic regression was 0.001.

**Table 1:** Body mass index in relation to socioeconomic characteristics among Bangladeshi women age 15–49 years

Variable	Underweight	Normal weight	Overweight and Obese
	< 18.5 kg/m^2^	18.5–24.9 kg/m^2^	≥25 kg/m^2^
Age (in years)			
15–19	2819 (32.0)	5440 (61.8)	544 (6.2)
20–29	6365 (22.7)	16,845 (60.1)	4800 (17.1)
30–39	4140 (18.7)	12,357 (55.9)	5599 (25.3)
40–49	3727 (22.8)	8708 (53.1)	3951 (24.1)
Place of Residence (Urban/Rural)			
Urban	4213 (16.0)	14,178 (53.9)	7931 (30.1)
Rural	12,838 (26.2)	29,172 (59.6)	6963 (14.2)
Highest Educational Level			
No education	6326 (30.4)	11,930 (57.3)	2553 (12.3)
Primary	5445 (24.5)	13,099 (59.0)	3671 (16.5)
Secondary	4721 (18.2)	14,976 (57.8)	6221 (24.0)
Higher	559 (8.8)	3345 (52.7)	2449 (38.6)
Parity			
No children	1772 (23.5)	4684 (62.2)	1072 (14.2)
1–2 children	7122 (20.7)	19,859 (57.7)	7423 (21.6)
3–4 children	4776 (21.6)	12,637 (57.1)	4737 (21.4)
5^+^ children	3381 (30.2)	6170 (55.0)	1662 (14.8)
Current Marital Status			
Married	15,513 (22.0)	40,803 (57.9)	14,103 (20.0)
Widowed	874 (31.0)	1422 (50.4)	526 (18.6)
Divorced	255 (30.6)	478 (57.4)	100 (12.0)
No longer living together/Separated	409 (33.5)	647 (53.0)	165 (13.5)
Wealth Index			
Poorest	4814 (36.2)	7538 (56.7)	936 (7.0)
Poorer	4057 (29.1)	8550 (61.3)	1339 (9.6)
Middle	2896 (18.5)	9360 (659.7)	3425 (21.8)
Richer	1717 (9.8)	8826 (50.1)	7064 (40.1)
Richest	1452 (10.5)	7108 (51.5)	5256 (38.0)
Year			
2004	3600 (32.2)	6399 (57.3)	1174 (10.5)
2007	3011 (27.4)	6329 (57.6)	1653 (15.0)
2011	3968 (22.4)	10,284 (57.9)	3497 (19.7)
2014	6472 (18.3)	20,338 (57.5)	8570 (24.2)

## Results

### Prevalence of underweight, overweight and obesity

Table 1 shows the descriptive statistics of weight status (body mass index) in relation to socio-demographic characteristics among women in Bangladesh age 15–49 years. Over the ten years period, the prevalence of underweight reduced by 43.2% (from 32.2%in 2004 to 18.3% in 2014) and the prevalence of overweight and obesity increased by 130.5% (from 10.5% in 2004 to 24.2% in 2014)The highest (32.0%) prevalence of underweight was among the youngest (15–19 years) age group of women while lowest (18.7%) prevalence was among the middle age group (30–39 years). On the other hand, the prevalence of overweight and obesity was highest (25.3%) among the middle age group(30–39 years) while lowest (6.2%) among the youngest age group (15–19 years). In the regional context, rural women were more prevalent (26.2%) of being underweight than urban women (16.0%). Conversely, urban women were more prevalent (30.1%) of being overweight and obese than rural women (14.2%). The highest prevalence of underweight was among women with no education (30.4%). On the other hand, women with higher education were most prevalent (38.6%) of being overweight and obese. The prevalence of overweight and obesity was highest (40.1%) among richer women whereas the prevalence of underweight was highest (36.2%) among poorest women.

The prevalence of underweight was highest (30.2%) among women who had 5 or more children. On the contrary, women having 1–2 children were most prevalent (21.6%) of being overweight and obese. Women who were separated from their husband were most prevalent (33.5%) of being underweight while married women had highest (20.0%) prevalence of being overweight and obese.

### Determinants of underweight, overweight and obesity

Multinomial logistic regression analysis demonstrated in Table 2 to show the association between BMI and socio-demographic characteristics of women. Characteristics such as age, educational status, wealth index and survey year were negatively associated with being underweight and positively associated with being overweight and obese. Moreover, rural women, parity, not being married were positively correlated with being underweight and negatively correlated with being overweight and obese.

**Table 2:** The association between underweight and overweight, and socioeconomic determinants of Bangladeshi women aged 15–49 years

Variable	Underweight< 18.5 kg/m^2^	Overweight and obesity≥25.0 kg/m^2^
Age (in years)		
15–19	1	1
20–29	0.7 (0.6–0.7)*	2.8 (2.5–3.1)*
30–39	0.5 (0.4–0.50)*	5.2 (4.7–5.8)*
40–49	0.5 (0.5–0.6)*	5.7 (5.1–6.4)*
Place of Residence (Urban/Rural)		
Urban	1	1
Rural	1.1 (1.1–1.2)*	0.7 (0.7–0.8)*
Highest Educational Level		
No education	1	1
Primary	0.9 (0.8–0.9)*	1.3 (1.2–1.3)*
Secondary	0.8 (0.7–0.8)*	1.6 (1.5–1.7)*
Higher	0.6 (0.5–0.6)*	1.7 (1.6–1.9)*
Parity		
No children	1	1
1–2 children	1.1 (1.1–1.2)*	1.1 (1.0–1.1)
3–4 children	1.2 (1.1–1.3)*	1.0 (0.9–1.1)
5+ children	1.7 (1.5–1.8)*	0.8 (0.7–0.9)*
Current Marital Status		
Married	1	1
Widowed	1.6 (1.4–1.7) *	0.9 (0.8–1.0)
Divorced	1.5 (1.3–1.8)*	0.6 (0.5–0.8)*
No longerLiving together/separated		
	1.6 (1.4–1.9)*	0.8 (0.6–0.9)
Wealth Index		
Poorest	1	1
Poorer	0.8 (0.7–0.8)*	1.2 (1.1–1.3)*
Middle	0.7 (0.6–0.7)*	1.7 (1.5–1.8)*
Richer	0.6 (0.5–0.6)*	2.4 (2.2–2.6)*
Richest	0.4 (0.3–0.4)*	4.5 (4.1–4.9)*
Year		
2004	1	1
2007	0.9 (0.8–0.9)*	1.4 (1.3–1.5)*
2011	0.7 (0.7–0.8)*	1.9 (1.7–2.0)*
2014	0.6 (0.5–0.6)*	2.5 (2.3–2.7)*

Results have been drawn from multinomial logistic regression analysis

The overall *P*-value for this model was 0.001

*Statistically significant (*p* < 0.001) odds ratio

Reference category for dependent variable was normal weight

Women age 20–29 years were less likely to be underweight (OR = 0.7, 95% CI: 0.6–0.7) whereas the likelihood of being overweight and obese was 5 times (OR = 5.7, 95% CI: 5.1–6.4) higher among women age 40–49 years compared to age group 15–19 years respectively. With regards to regional distribution, rural women were less likely to be overweight and obese (OR = 0.7, 95% CI: 0.7–0.8) while more likely to be underweight (OR = 1.1, 95% CI: 1.1–1.2) relative to urban women. Compared to women with no education, women with higher education were less likely to be underweight (OR = 0.6, 95% CI: 0.5–0.6) and more likely to be overweight and obese (OR = 1.7, 95% CI: 1.6–1.9). The odds of overweight and obesity was lower (OR = 0.8, 95% CI: 0.7–0.9) among women having 5or more children compared to women having no children. Similarly, divorced women were less likely to be overweight and obese (OR = 0.6, 95% CI: 0.5–0.8) compared to married women. The likelihood of being overweight and obese was 4.5 times (95% CI: 4.1–4.9) higher among women who were in richest quintile compared to poorest women. They were also less likely to be underweight (OR = 0.4, 95% CI: 0.3–0.4) relative to same reference category.

## Discussion

The present study articulates that the prevalence of being overweight and obese is considerably increasing (from 10.51% in 2004 to 24.22% in 2014) whereas the prevalence of underweight is decreasing (from 32.22% in 2004 to 18.29% in 2014) gradually among ever-married women in Bangladesh. These findings are consistent with another studies conducted in Bangladesh [22, 31] as well as several other countries [7, 32, 33]. This study also met the prediction that the over nutrition will exceed the undernutrition by year 2015 [34, 35]. Although the prevalence of underweight is decreasing, it is still very high. This situation of co-existence of underweight and overweight/obesity within same population is called double burden of malnutrition [3, 5, 6]. Many other developing countries are facing such situation of double burden of malnutrition [6, 33, 34, 36, 37]. Based on the current study, urban women are more prevalent of being overweight and obese than rural women. The prevalence of being overweight and obese in developing countries has association with rising urbanization [7]. In urban areas, residents have available access on technologies requiring less energy, availability of energy-dense food, modern transportation system, limited space for physical activity, sedentary lifestyle-all promote the higher prevalence of being overweight and obese in urban areas [4, 38, 39]. The findings of the study have explored that the older women are more likely to be overweight and obese than their younger counterpart. This study concurs with the many other studies [31, 40]. Similar result was found in the study of Ethiopia [41] and India [32] suggesting that the reduced level of physical activity and increased intake of energy-dense foods by women increases with age. Fat free mass decreases and fat mass increases in body composition after 30 years of age [42, 43] might be the possible explanation. The findings of this study revealed that the prevalence of being overweight and obese is significantly higher among higher educated women [31, 40, 41]. This outcome is consistent with the other study of developing countries [44]. The reason behind this may be the consequence of switches from manual work to more sedentary occupation of higher educated women which in turn decline in physical activity. Moreover, a study has found that educational level is positively related to leisure time and negatively related to work index (index of habituated physical activity) [45]. Women in the richest quintile have more possibility to be overweight and obese which are consistent with the several similar studies of Bangladesh [23, 40]. Subramanian et al. (2011) confirmed that there is a positive relationship between wealth and overweight in India [46]. Possible explanation for this, in developing countries, with changes in income, the dietary behavior is also changes like consume more energy-dense foods and lead a sedentary lifestyle which in turn them into more likely to be overweight and obese. Pingali (2007) have found that due to globalization, urbanization, rapid economic and income growth, Asian diets are shifting dramatically towards Western diet which is characterized by high protein and energy dense food [47]. Women having five or more than five children are less likely to be overweight and obese compared to the women who have no children. Another study in Bangladesh demonstrated that women with fewer children (< 3) are more likely to be overweight [24]. Since rearing child is one of the major responsibility of women in developing countries like Bangladesh, upbringing of many children at a time affect mother’s health considerably. Compared to married women, divorced women are less likely to be overweight and obese and more likely to be underweight. Several relevant studies revealed that overweight and obesity are more prevalent among married women [30, 41, 47]. Women in developing countries like Bangladesh are usually dependent on their husbands. Getting divorce may make them insecure both in financial and social issues that could be responsible for women’s mental and physical hazards.

This study has several strengths. Firstly, the data used in this study were extracted from large nationally representative surveys conducted at several time points. Secondly, the response rate among all eligible women was excellent (92 to 97.9%) in all four surveys. Thirdly, anthropometric data (height and weight) were collected by trained personnel using same measurement equipment which made possible to compare data at different time points. There are also several limitations that need to be considered in future studies. Datasets lacked some important determinants of the dependent variable such as food habit, physical activity of women which could have helped to understand the relationship between selected independent variable and BMI. The surveys did not collect data on abdominal and waist-to hip circumference which could have explained abdominal obesity. Confounding effect has not been checked for the multinomial logistic regression analysis of this study.

## Conclusion

Double burden of malnutrition is now emerging nutritional phenotype for ever-married women in Bangladesh. Moreover, overweight and obesity has been tremendously rising but simultaneously underweight did not fall significantly over the 10-years period. Age, Place of residence (Urban/Rural), educational status, parity, marital status, wealth index, and year are significantly associated with both overweight/obesity and underweight problems among reproductive age women in Bangladesh. This study suggests that strategies for preventing both underweight and overweight/obesity simultaneously among reproductive women need to be implemented considering regional context and their socioeconomic status (SES). Although, it is a great challenge to address interventions for combating both extreme form of malnutrition, viz. underweight and overweight/obesity at a time, programs should be taken technically to prevent both types of problem. Also, raising awareness about healthy life may be helpful to prevent both underweight and overweight/obesity. More in depth researches are required to take appropriate strategies to handle such type of paradoxical situation in Bangladesh.

## Abbreviation

BDHS: Bangladesh Demographic and Health Survey
BMI: Body mass index
CDs: Communicable diseases
IOTF: International Obesity Task Force
MOHFW: Ministry of Health and Family Welfare
NDCs: Non-communicable diseases
NIPORT: National Institute of Population and Research
PHC: Population and Housing Census
SES: Socioeconomic Status
